# Composite paraganglioma-ganglioneuroma in the retroperitoneum

**DOI:** 10.1186/1477-7819-7-81

**Published:** 2009-11-05

**Authors:** Shoji Hirasaki, Hiromitsu Kanzaki, Masato Okuda, Seiyuu Suzuki, Tetsuji Fukuhara, Toshihito Hanaoka

**Affiliations:** 1 Division of Gastroenterology, Sumitomo Besshi Hospital, 3-1 Ohji-cho, Niihama 7928543, Japan; 2 Division of Gastroenterology, Kubo Hospital, 1-1-19 Uchibori, Imabari 7992116, Japan; 3 Department of Surgery, Sumitomo Besshi Hospital, 3-1 Ohji-cho, Niihama 7928543, Japan

## Abstract

**Background:**

Paragangliomas occur most commonly in head and neck region and much less frequently, they are found in the retroperitoneum. Composite paraganglioma-ganglioneuroma of the retroperitoneum is very rare.

**Case presentation:**

We present an unusual case of retroperitoneal composite paraganglioma-ganglioneuroma discovered on computed tomography in a 63-year-old female patient. Routine hematological examination and biochemical tests were within normal limits. Plasma adrenaline was 0.042 ng/ml, plasma noradrenaline 0.341 ng/ml, and plasma dopamine <0.01 ng/ml. An abdominal contrast-enhanced CT scan and magnetic resonance imaging revealed a 6.5 cm heterogeneous retroperitoneal mass with a cystic component. The retroperitoneal tumor accumulated ^131^I-Metaiodobenzylguanidine (^131^I-MIBG) 48 hours after radioisotope injection. Under the diagnosis of paraganglioma in the retroperitoneum, the patient underwent surgery. The resected tumor (6.5 × 5 × 3 cm) was solid and easily removed en bloc. The cut surface of the tumor and histology revealed two different components in the tumor: paraganglioma centrally and ganglioneuroma on the periphery. She remains disease-free 18 months after surgery.

**Conclusion:**

This case reminds us that neuroendocrine tumor should be included in the differential diagnosis of a retroperitoneal mass although composite paraganglioma-ganglioneuroma in the retroperitoneum is very rare.

## Background

Pheochromocytomas arising from outside the adrenal glands are called paragangliomas [1] and constitute a relatively rare disease, arising from undifferentiated cells of the primitive neural crest [2,3]. Paragangliomas occur most commonly in head and neck region [4], and much less frequently, they are found in the retroperitoneum [3]. Extraadrenal paragangliomas, which account for 5-10% of these tumors, are found in the retroperitoneum, thorax, and urinary bladder [5]; composite tumor of the retroperitoneum is rare. To our knowledge, at least 4 cases of composite paraganglioma-ganglioneuroma in the urinary bladder have been reported [5-7]; however, there have been only few reports of retroperitoneal composite paraganglioma-ganglioneuroma. Herein, we report an unusual case of composite paraganglioma-ganglioneuroma in the retroperitoneum successfully treated with surgery.

## Case presentation

**Figure 1:**
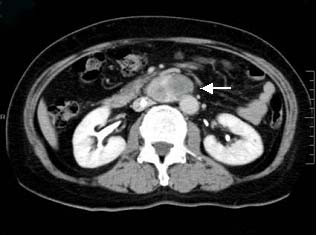
**An abdominal contrast-enhanced CT scan incidentally demonstrated a heterogeneous mass, 6.5 cm in diameter, with cystic lesion in the retroperitoneum**. The tumor exhibited marked enhancement after intravenous administration of contrast material.

**Figure 2:**
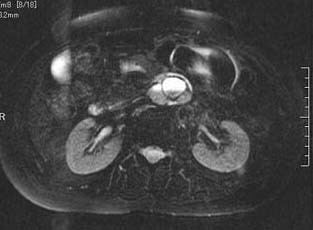
**T2-weighted magnetic resonance images revealed a high intensity tumor those signal intensity was greater than that of the liver with central higher signal intensity**.

**Figure 3:**
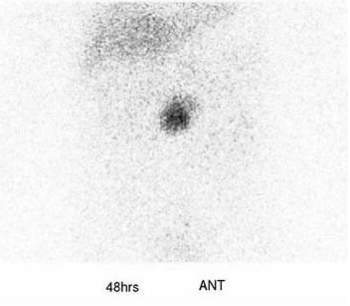
**^131^I-Metaiodobenzylguanidine (^131^I-MIBG) scintigraphic scan tomography showed ^131^I-MIBG accumulation in the retroperitoneal tumor**.

**Figure 4:**
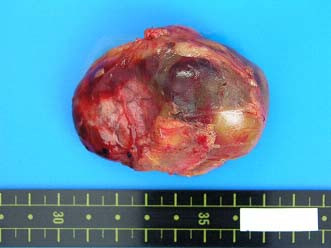
**Macroscopic finding of the tumor**. The resected tumor, 6.5 × 5 × 3 cm, was encapsulated on its external surface.

**Figure 5:**
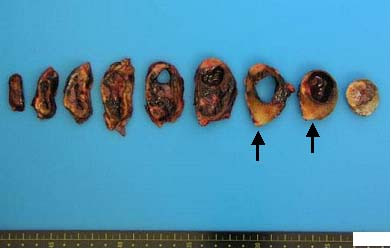
**The cut surface showed two different components including a brown-red lesion with a cyst and a whitish lesion (arrows)**.

**Figure 6:**
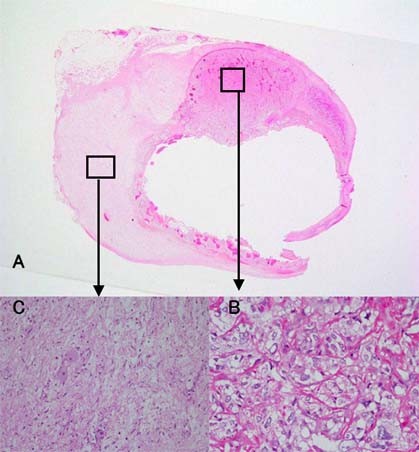
**Microscopic findings of the tumor**. (A) Two different components were recognized in the tumor. (B) Histological examination of the brown-red lesion with a cyst demonstrating a nest of neoplastic cells (Zellballen) containing finely granular cytoplasm and enlarged nuclei. (HE stain × 200). (C) The whitish lesion revealed the intersecting bundles of Schwann cells contained scattered ganglion cells. The ganglion cells had plump cytoplasm (HE stain × 100).

The patient was a 63-year-old-woman, 158 cm tall and weighing 62 kg. She was admitted to our hospital for left femoral shaft fracture. She underwent an abdominal contrast-enhanced CT scan because she developed left leg edema and was suspected of suffering deep vein thrombosis of the lower extremity, and a large retroperitoneal mass was discovered. She had no abdominal symptoms, had been in good health, and had no specific family, past medical, or drug history. She had not complained of headache and had no history of hypertension. Routine hematological examination and biochemical tests were within normal limits. Plasma adrenaline was 0.042 ng/ml [normal range (NR): <0.07], plasma noradrenaline 0.341 ng/ml (NR: 0.06-0.46), and plasma dopamine <0.01 ng/ml (NR: <0.014). Regarding tumor markers, carbohydrate antigen 19-9, carcinoembryonic antigen (CEA) and alpha fetoprotein (AFP) were all negative. An abdominal contrast-enhanced CT scan (Fig. 1) and magnetic resonance imaging (MRI) (Fig. 2) revealed a 6.5 cm heterogeneous retroperitoneal mass with a cystic component. MRI showed a high intensity tumor with signal intensity greater than that of the liver and central higher signal intensity on T2-weighted images. ^131^I-Metaiodobenzylguanidine (^131^I-MIBG) scintigraphic scan tomography was performed and the retroperitoneal tumor accumulated ^131^I-MIBG 48 hours after radioisotope injection (Fig. 3). Her deep vein thrombosis seemed to be caused by congestion and movement limit due to bone fracture and had no connection with retroperitoneal mass because her leg edema improved before abdominal surgery. Under the diagnosis of paraganglioma in the retroperitoneum, the patient underwent surgery. The resected tumor (6.5 × 5 × 3 cm) was solid and easily removed en bloc. The tumor appeared grossly encapsulated on its external surface (Fig. 4). The cut surface showed two distinct components: brown-red lesion with a cyst and a whitish area accounting for 10% of the tumor (Fig. 5, Fig. 6A). The tumor had no necrosis or hemorrhage. Histological examination of the brown-red lesion with a cyst revealed well-defined nests of neoplastic cells containing finely granular cytoplasm and enlarged nuclei referred to as 'Zellballen' (Fig. 6B). Several tumor cells had eosinophilic hyaline globules in the cytoplasm. These neoplastic cells were positive for chromogranin A, synaptophysin and Grimelius stain. A population of S-100-positive sustentacular cells that were present at the peripheries of the cell nests was seen. This lesion was diagnosed as paraganglioma. Paraganglioma had <1% of MIB-1-positive cells. Histological examination of the whitish area revealed the proliferation of spindle shaped Schwann cells and a small number of ganglion cells, characterized by plump cytoplasm (Fig 6C). Immunohistochemical staining revealed positive immunoreactivity for S-100 protein in these cells. Cells of each component were well differentiated and no mitosis was observed. There was no evidence of malignancy. The tumor was composed of approximately 90% paraganglioma and 10% ganglioneuroma. Focal interminglings of the two components were noted; however, two components were separated in the greater part of the tumor. Abrupt transition of two different components of paraganglioma and ganglioneuroma was seen. The diagnosis, based on these histological findings, was composite paraganglioma-ganglioneuroma in the retroperitoneum. The postoperative course was uneventful and no additional therapy was given according to informed consent. She has been under close periodic observation and there is no evidence of this disease 18 months after surgery.

## Discussion

The paraganglia are widely dispersed collections of specialized neural crest cells that lie adjacent to the sympathetic ganglia and plexuses throughout the body. The paraganglionic system includes the adrenal medulla, the chemoreceptors, vagal body, and small groups of cells associated with the thoracic as well as intraabdominal and retroperitoneal ganglia [8]. Tumors that arise from chromaffin cells of the adrenal medulla are called pheochromocytomas, whereas those that occur in paraganglia at other sites are referred to as paragangliomas [9]. Ganglioneuroma is a benign neoplasm composed of Schwann cells and ganglion cells. The most affected anatomical sites are the posterior mediastinum, retroperitoneum, adrenal gland and head and neck soft tissue [10]. Solitary ganglioneuromas most commonly occur in infants and young children, slightly more often in girls than boys, with a female-to-male ratio of about 3:1 [11]. The majority is diagnosed before the patient is 10 years of age.

Paraganglioma shows marked contrast enhancement on contrast-enhanced CT scan [12,13]. MRI can be used to help to locate a paraganglioma; however, only about 80% of T2-weighted MRI studies will show the characteristic uniform high-signal-intensity image because the presence of internal hemorrhage may reduce signal intensity [9]. In the present case, diagnostic images obtained by contrast-enhanced CT scan, MRI and ^131^I-MIBG scintigraphic scan tomography were compatible with paraganglioma. But we could not diagnose this tumor as composite paraganglioma-ganglioneuroma.

Paraganglioma-ganglioneuroma is a rare composite tumor, and at least one case of extra-adrenal retroperitoneal ganglioneuroma-pheochromocytoma has been reported [14]. Usuda et al reviewed 4 patients with paraganglioma-ganglioneuroma in the urinary bladder [6]. All 4 patients were older than 40 years of age (range, 49-81 years), 3 of 4 patients with composite tumor were women, and 2 patients had a symptom of headache. They reported that it was difficult to predict the clinical behavior of composite tumors. These 4 reported cases of composite paraganglioma-ganglioneuromas in the urinary bladder showed no malignant features such as extra-bladder infiltration or metastasis. The natural history of composite paraganglioma-ganglioneuromas remains unclear. Thus, accumulation of long-term follow-up cases may provide valuable prognostic information on this composite tumor. The pathogenesis of composite paraganglioma-ganglioneuroma also remains unknown. Although we could not make a conclusive remark, we thought that the tumor might be a collision tumor because two components (paraganglioma and ganglioneuroma) were separated in the greater part of the tumor and transitional zone was not recognized in the present case.

It is not possible to differentiate benign from malignant paraganglioma confidently with imaging alone. With the exception of the presence of distant metastases, there are no absolute criteria for malignancy in paragangliomas. However, features more frequently noted in malignant tumors are extraadrenal location, greater tumor weight, confluent necrosis, and the presence of vascular invasion and/or extensive local invasion [5]. According to Hayes et al [15], malignant paragangliomas tend to be large and their four cases had average diameter of 13.8 cm (range, 10.7-17.0). Thus a tumor size of larger than 10 cm may be indicative of malignant behavior. Assessing the proliferative index seems to be important, because all of the analyzed benign tumors showed <1% of MIB-1-positive cells [16].

In the present case, there were no histological features suggesting malignancy, and MIB-1-positive cells were <1%; however, we should be attentive to recurrence in this case. Sclafani et al found that 11 of 22 (50%) retroperitoneal paragangliomas metastasized to distant organs [17] and 2 of 11 (18%) patients developed their first metastases more than 7 years after diagnosis; thus, a longer follow-up is needed for this disease.

Retroperitoneal paraganglioma is treated in principle by surgical resection. Composite paraganglioma-ganglioneuroma in the retroperitoneum is very rare. However, as probably most of the retroperitoneal paragangliomas are benign, it is likely that there are patients with latent retroperitoneal paraganglioma, which may be incidentally discovered in the future as a result of advances in diagnostic imaging, such as improved CT and MRI.

## Conclusion

Although composite paraganglioma-ganglioneuroma in the retroperitoneum is not common, the possibility of a neuroendocrine tumor should be considered in the differential diagnosis of a retroperitoneal mass. When a large retroperitoneal mass is incidentally found by diagnostic imaging, it should be carefully examined and resection should be considered, especially in patients with tumor size of larger than 10 cm.

## Consent

Written informed consent was obtained from the patient for publication of this case report and accompanying images. A copy of the written consent is available for review by the Editor-in-Chief of this journal.

## Competing interests

The authors declare that they have no competing interests.

## Authors' contributions

SH and HK reviewed the literature and wrote the case presentation, SH, HK, MO, SS were involved in the care of the patient, TF and TH performed surgical management. All authors read and approved the final manuscript.
